# Using value of information to guide evaluation of decision supports for differential diagnosis: is it time for a new look?

**DOI:** 10.1186/1472-6947-13-105

**Published:** 2013-09-11

**Authors:** R Scott Braithwaite, Matthew Scotch

**Affiliations:** 1 Department of Population Health, New York University School of Medicine, 550 First Avenue, VZ30 6th floor, 615, New York, NY 10016, USA; 2 Department of Biomedical Informatics, Arizona State University, Scottsdale, AZ, USA

**Keywords:** Value of information, Decision support, Differential diagnosis

## Abstract

**Background:**

Decision support systems for differential diagnosis have traditionally been evaluated on the basis of criteria how sensitively and specifically they are able to identify the correct diagnosis established by expert clinicians.

**Discussion:**

This article questions whether evaluation criteria pertaining to identifying the correct diagnosis are most appropriate or useful. Instead it advocates evaluation of decision support systems for differential diagnosis based on the criterion of maximizing value of information.

**Summary:**

This approach quantitatively and systematically integrates several important clinical management priorities, including avoiding serious diagnostic errors of omission and avoiding harmful or expensive tests.

## Background

In clinical care, there has been much effort on decreasing medical errors including diagnostic errors of omission (DEO). In fact, DEOs account for a large proportion of medical adverse events and form the second-leading cause for malpractice suits against hospitals [1]. Improving differential diagnostic (DDX) decision support tools has great potential to reduce DEOs [2-4]. However, DDX decision support tools are often constructed with the goal of identifying a single correct diagnosis, which does not necessarily diminish DEOs unless the tool has optimal performance characteristics. This article discusses whether an alternative criterion for evaluating DDX tools, in particular maximization of value of information [5], might yield DDX tools that are more effective at reducing DEOs [1,6], than ones designed to detect the best diagnosis [4,7-11].

## Discussion

### The value of DDX tools

Decision support tools exist to facilitate better decisions, and better health decisions are those choices that minimize morbidity and mortality, in concordance with patient preferences and principles of shared decision making [12]. Accordingly, value of information (VOI) may be a particularly suitable framework for evaluating DDX decision support tools. This is because it considers the monetarized benefit of morbidity and mortality that can be prevented through improvements in decisions that are made possible by new information, after considering the costs and harms of obtaining that information. In the context of DDX decision support tools, applying VOI can be viewed as a quantitative means of integrating several desirable goals. These include maximizing the morbidity and mortality of DEOs that may be averted by DDX tools, and minimizing the incremental costs and harms from the diagnostic tests that these tools may induce.

### Brief summary of VOI

VOI is a framework developed by Claxton [5,13] that has its conceptual roots in decision analysis and economics. A detailed description of VOI is beyond the scope of this paper. However, VOI assessment of an informatics intervention can be viewed as a three-step mathematical calculation: (1) “How would health outcomes change because of different decision making that would result from using the intervention?”, (2) “What is the monetarized value of that change in health outcomes?” and (3) “How does #2 change after considering the costs of the intervention and considering the downstream consequences of its use?” Accordingly, if health outcomes would be improved by the post-intervention decision making compared to the pre-intervention decision making, (question 1), the expected value of information (EVI) would be numerically higher (or less negative) (question 2). However, if incremental costs of using the intervention are greater than those of not using the intervention including differences in diagnostic tests ordered and their downstream consequences (including false positives and complications), then the EVI would be numerically lower or more negative (question 3).

### Illustrative scenario

Consider a 64 year-old man who presents to an emergency department with severe chest pain but without dyspnea or other pain. While there are literally hundreds of potential diagnoses that could be considered by a DDX tool, the tool is most clinically useful if it initially restricts its attention to the subgroup of those diagnosis that are “actionable”. Here, “actionable” refers to a situation in which rapidly identifying a particular diagnosis could lead to decisions that would improve morbidity and mortality. Conversely, delaying the identification of that diagnosis would lead to decisions that worsened morbidity and mortality. This definition is similar to Ramnarayan’s definition of “clinically relevant diagnoses” [1] but can be specified in terms of morbidity and mortality, and therefore is more closely linked to VOI. (“Actionability” can be defined as the expected value (EV) of rapid treatment minus the EV of delayed or no treatment. It is a distinct concept from “import” [14], which is an approximation of the true positivity of a test with respect to a disease in the differential diagnosis, and does not necessarily contain any information about the incremental morbidity and mortality that may be prevented by a timely diagnosis.) For example, diagnoses of myocardial infarction, pulmonary embolism, dissecting aortic aneurysm, and pericarditis would be highly actionable because of the morbidity and mortality burden that could be mitigated through prompt action. On the other hand, diagnosis of non-dyspneic pleuritis would be less actionable because potential mechanisms of pleuritis (*e.g.* malignancies), while capable of causing serious morbidity and mortality, would not necessarily lead to great reductions in morbidity and mortality by quick identification and response. Similarly, other possible diagnoses such as panic attacks, neuralgic pain from zoster or pain from costochrondritis would be even less actionable, unless it dramatically reduced the probability of an actionable diagnosis through considerations of physiological incompatibility.

### Relationship between VOI criteria and DDX decision support tools

**Figure 1:**
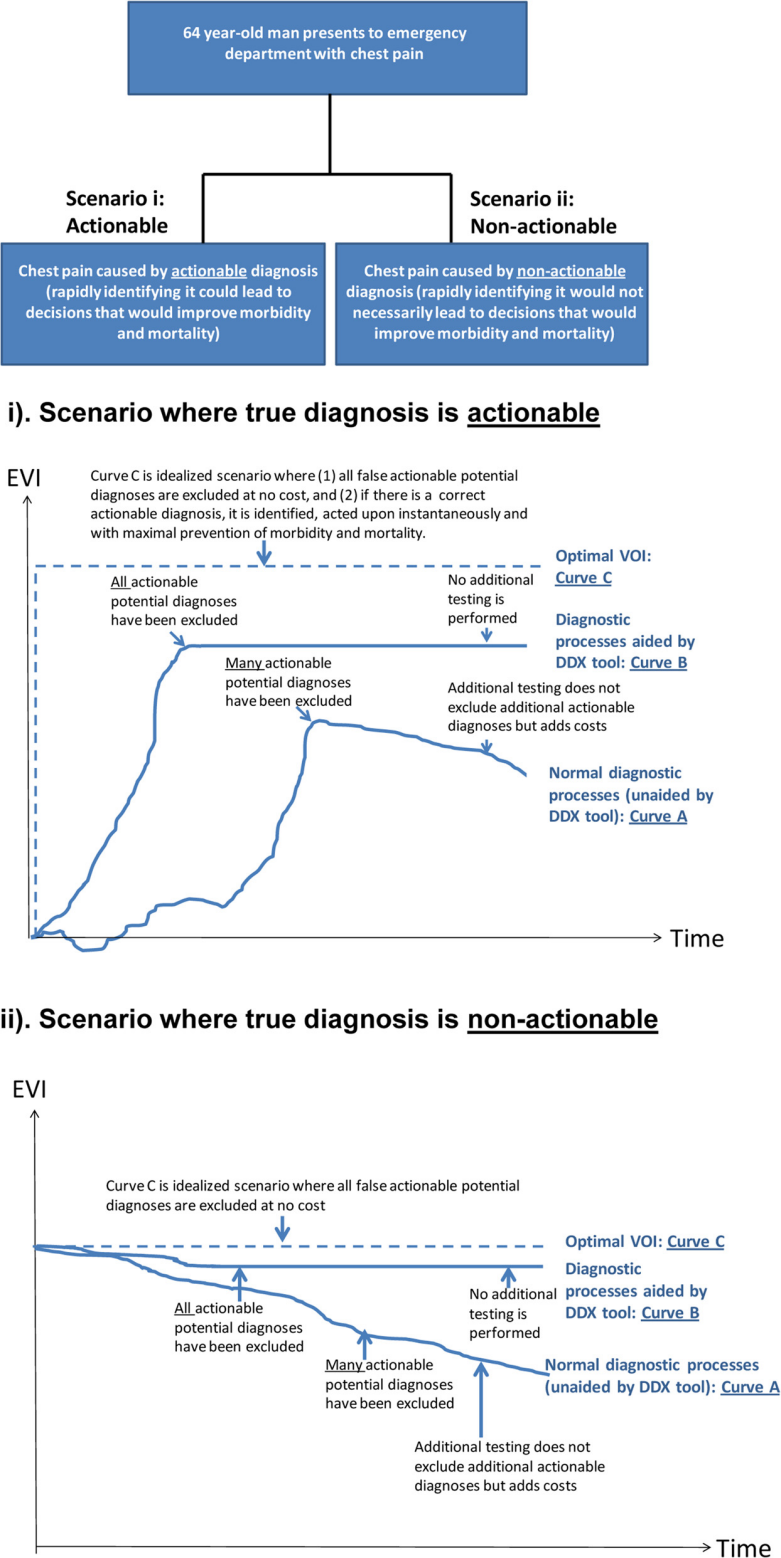
**Scenarios where true diagnosis is actionable (i) versus inactionable (ii).** “Actionability” refers to the idea that rapid identification can reduce preventable morbidity and mortality compared to delayed identification. When the true diagnosis is actionable **(scenario “i”)**, the perfect diagnostic strategy instantaneously actuates the increment in EVI made possible by ascertaining that diagnosis, at minimal cost. When the true diagnosis is inactionable **(scenario “ii”)**, the perfect diagnostic strategy minimizes decrements in EVI associated with ruling out other diagnoses because of harms and/or costs associated with diagnostic tests. EVI: expected value of information.

**Figure 2:**
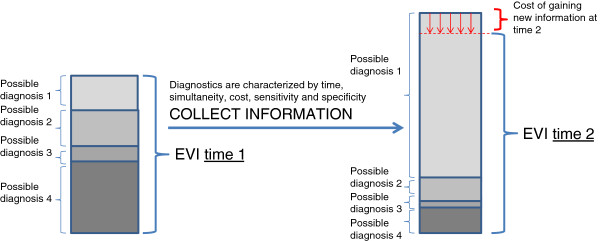
**The expected value of information of differential diagnostic tools.** The EVI of DDX tools is based on the monetarized value of the morbidity and mortality prevented by promptly establishing a particular diagnosis, multiplied by the probability of that diagnosis, minus costs and monetarized value of time and complications from diagnostic tests that modify the probability of that diagnosis. The prior diagnostic possibilities (left column) are updated by diagnostic tests to produce updated diagnostic probabilities (right column). The updating here increases the likelihood of those diagnoses with greater preventable morbidity and mortality, therefore increasing EVI. However, the updating could proceed in the opposite direction, increasing the likelihood of diagnoses with lesser preventable morbidity and mortality, therefore, decreasing EVI. Costs associated with the diagnostic tests lower EVI (shown here by the red arrows). An optimal DDX decision support tool could be viewed as that which maximizes EVI. EVI: expected value of information.

Consider two similar scenarios in which the 64 year-old man described above presents to the emergency department with chest pain, one of which is caused by a diagnosis that is extremely actionable (Figure 1, scenario i), and another that is caused by a diagnosis that is only slightly actionable (Figure 1, scenario ii). With both scenarios, a more clinically useful DDX tool would more rapidly maximize increments in EVI. In the first scenario, in which the chest pain has an actionable diagnosis, the tool would guide decision making to rapidly accurately identify the actionable cause of chest pain, thereby leading to the improvements in morbidity and mortality that could elevate EVI (Figure 1, scenario i). In the second scenario, in which the chest pain does not have an actionable etiology (Figure 1, scenario ii), the tool would guide decision making to rapidly and accurately rule-out the actionable causes of chest pain (Figure 1, scenario ii), or, if feasible, to rapidly rule-in a non-actionable diagnosis that is incompatible with the actionable diagnoses. As evaluated by EVI, better DDX decision support tools would be those that lead to clinical management strategies that avoid diagnostic resources that do not end up reducing preventable morbidity and mortality. Because not all diagnostic tests can occur simultaneously, the ideal DDX decision strategy tool will guide ordering of diagnostic tests to maximize EVI, and may even sometimes increase EVI fastest by ruling-in a common diagnosis with lower preventable morbidity and mortality that is incompatible with a rarer diagnosis with higher preventable morbidity and mortality (Figure 2).

### Limitations of prior efforts to use VOI to evaluate DDX

Downs et al. (1997) first proposed applying a VOI to evaluate DDX processes [15]. However, their approach had multiple limitations which our current approach improves upon, and therefore our approach may be more feasible. First, they calculated VOI using utilities that were not elicited using standard decision analytic approaches for health states (e.g., time tradeoff) [16], and consequently their VOI calculations did not necessarily reflect potential morbidity and mortality improvement. For example, viral pneumonia correctly diagnosed was given a higher utility than bacterial pneumonia correctly diagnosed, even though correct diagnosis of bacterial pneumonia would be expected to improve quality and quantity of life, and therefore to improve health-state based utility, much more than correct diagnosis of viral pneumonia. In contrast, we suggest using health state-based utilities, which comport more readily with applications of VOI to medical decision making.

Second, their approach yielded the unrealistic result that the VOI of most information-seeking was zero because they did not quantify the down-side to additional information gathering: time, patient discomfort, and complications. All of these would be expected to lead to a negative VOI even when no informative diagnostic information is produced. Although they attempted to address the limitation subsequently [17] by modifying their VOI algorithm to “calculate the average of the expected utility across all the diagnoses, then subtract the expected utility of the diagnosis with the highest utility”, this modification presumes that VOI reflects changes in the EV of particular diseases more directly than changes in the EV of the most favored decision, unlike subsequent uses of VOI [5]. In contrast, we recommend valuating negative as well as positive consequences of additional information gathering, and we advocate using VOI based on the EV of the most favored decision [5].

Third, their approach does not identify a point at which the diagnostic process should naturally stop, which is a concept with great clinical value. If most of the information comprising the diagnostic process has an EVI of zero, the diagnostic process could go on indefinitely. In contrast, because we consider negative EVIs, a decline in the EVI curve would be expected to occur at some point (for example, when clinicians are adopting “shotgun” diagnostic strategies that may have side effects), signaling that the diagnostic phase should conclude.

### Possible VOI metrics for assessing performance of DDX decision support tools

An ideal diagnostic process would minimize preventable morbidity and mortality to the patient, and would also minimize cost, time, and discomfort. The value of EVI at the end of the diagnostic process is a candidate for distinguishing a more favorable diagnostic trajectory from a less favorable diagnostic strategy and clinical management. For example, compare the EVI endpoint for an optimal diagnostic pathway (Curves C in Figure 1) compared to the EVI endpoint for typical, unaided diagnostic pathway (Curves A in Figure 1). A DDX decision support tool that leads to a greater proportional reduction, and therefore raises the EVI endpoint of Curve A closer to that of Curve C, would be preferred to a DDX tool that leaves the VOI endpoint of Curve A more distant from Curve C. Alternatively, area under the curve EVI might also be a suitable metric for assessing the performance of DDX tools. It is important to note that simply ordering all tests simultaneously to get rapid results would not optimize an EVI endpoint, because the resulting monetary cost, side effects, and time of these tests would lower the EVI trajectory.

### Limitations of VOI-optimized DDX tools

A DDX decision support tool optimized using VOI criteria would need to consider whether it is feasible to address more than one actionable diagnosis simultaneously, and this answer might vary for logistical reasons (e.g. number of operational CT scanners) between settings, institutions, and even between different shifts at the same institutions. For example, a DDX tool would need to factor in the possibility that it may be impossible to rule-out a myocardial infarction and a dissecting aortic aneurysm at a particular facility the same time, and therefore the DDX tool would ideally have the capability to guide prioritization of diagnostic decisions in accord with their relative increase in EVI. Further limitations of VOI-optimized DDX tools include an absence of a clear path towards enhancing shared decision making or considering individual patient preferences, and a potential lack of engagement with the types of cognitive errors and biases that lead to suboptimal decision making in the first place [18].

## Summary

Whether a DDX decision support tool gets the diagnosis right may not be as important as whether the tool is able to reduce preventable morbidity and mortality by reducing DOEs. VOI analyses in general, and EVI trajectories in particular, may be useful metrics for evaluating whether a DDX tool is able to pursue the important objective of reducing DOEs as well as other important objectives pertaining to minimizing harm and unnecessary expenditures.

## Competing interests

Both authors declare that they have no competing interests.

## Authors’ contributions

RSB made substantial contributions to conception and design and drafted the manuscript. MS made substantial contributions to conception and design and revised the manuscript critically for important intellectual content. All authors read and approved the final manuscript.

## Pre-publication history

The pre-publication history for this paper can be accessed here:

http://www.biomedcentral.com/1472-6947/13/105/prepub
